# Dendritic cells inhibit myeloid-derived suppressor cells infiltration and function in mouse B16 melanoma microenvironment

**DOI:** 10.1186/2051-1426-1-S1-P195

**Published:** 2013-11-07

**Authors:** Siyuan Xia, Jun Wei, Jingya Wang, Liqing Zhao, Zhinan Yin

**Affiliations:** 1 State Key Laboratory of Medicinal Chemical Biology, College of Life Sciences, Nankai University, Tianjin, China

## 

Roles of dendritic cells (DCs) in tumor immune responses have been reported to be very complex. It has been shown that in some tumor types, dendritic cells play an protective role by activating anti-tumor cells, while in other tumor types, they accelated tumor growth by inducing immune suppressive cells. Myeloid-derived suppressor cells (MDSCs) have also been proved to take part in immunoregulatory network in tumor microenvironment. Recently MDSCs have been found to impair the quality of DCs vaccine and promote Treg function in some tumors. However, whether and how DCs influence on MDSCs in tumor remain unclear. Thus we used CD11c-DTR mice to built a three week long DCs depletion model to find out their effect on MDSCs. DCs depletion accelated tumor growth. After three weeks, we isolated tumor infiltrating lymphocytes and analysed MDSCs and relative lymphocytes by flow cytometry. We found that depletion of CD11c+ DCs in mice B16 melanoma model led to MDSCs percentage increased in tumor microenvironment. In addition, CD4+ and CD8+ T cells produced less interferon-gamma and regulatory T cell (Treg) percentage increased. Thus, our results suggested that in cancer immunotherapy, DCs may inhibit MDSCs infiltration into tumor site and their function on T cells.

